# Community-based HIV Self-testing for Persons Who Use Drugs Can Contribute to Reaching Ending the HIV Epidemic in the US (EHE) Goals

**DOI:** 10.1093/ofid/ofae189

**Published:** 2024-06-17

**Authors:** Sabrina A Assoumou, Haley V Bonilla, Glorimar Ruiz-Mercado, Meg Von Lossnitzer, Richard Baker, Natalie D Crawford, Judith A Bernstein

**Affiliations:** Section of Infectious Diseases, Department of Medicine, Boston Medical Center, Boston, Massachusetts, USA; Department of Medicine, Boston University Chobanian & Avedisian School of Medicine, Boston, Massachusetts, USA; Section of Infectious Diseases, Department of Medicine, Boston Medical Center, Boston, Massachusetts, USA; Section of Infectious Diseases, Department of Medicine, Boston Medical Center, Boston, Massachusetts, USA; Mobile Prevention Team & Victory Connector, Victory Programs, Inc., Boston, Massachusetts, USA; Mobile Prevention Team & Victory Connector, Victory Programs, Inc., Boston, Massachusetts, USA; Department of Behavioral, Social, and Health Education Sciences, Rollins School of Public Health, Emory University, Atlanta, Georgia; Department of Community Health Sciences, Boston University School of Public Health, Boston, Massachusetts, USA

**Keywords:** HIV, HIV self-testing, HIV testing, Persons who use drugs, Persons who use opioids, HIV pre-exposure prophylaxis

## Abstract

In a pilot study providing HIV self-testing to persons who use drugs (N = 40), we identified 3 new HIV cases when partnering with a community-based organization. Most (82%) participants were interested in preexposure prophylaxis. HIV self-testing could contribute to efforts to Ending the HIV Epidemic in the United States.

**ClinicalTrials.gov registration:** NCT05528562

The US overdose crisis has led to outbreaks of HIV among persons who inject drugs (PWID) [1]. In 2019, the US Department of Health and Human Services declared a goal of Ending the HIV Epidemic (EHE) by 2030 [2]. This approach includes 4 pillars: diagnosing individuals with HIV as early as possible, providing access to treatment, preventing new HIV transmissions, and quickly responding to HIV clusters. HIV self-testing (HIVST) is recommended by the Centers for Disease Control and Prevention and could contribute to efforts to reach EHE goals [3]. When HIVST was first approved by the US Food and Drug Administration in 2012, it was hailed as a potential “game-changer” [4]. Unfortunately, HIVST has remained underused, especially among PWID [5]. Given that the COVID-19 pandemic increased the public's familiarity with home self-testing, an opportunity exists to increase HIVST uptake, especially for PWID.

Data show that individuals are more likely to follow recommendations from trusted sources [6]. Therefore, partnering with community-based organizations with existing relationships with PWID could increase the adoption of prevention methods such as HIVST. We collaborated with a community organization to provide free HIVST kits to persons who use drugs and safe spaces for testing. We determined the feasibility and acceptability of this approach as well as interest in HIV preexposure prophylaxis (PrEP) among participants.

## METHODS

We conducted a cross-sectional pilot study to examine the feasibility and acceptability of providing HIVST in low-threshold and trusted settings caring for PWID. The study was implemented at 2 Boston Victory Programs, Inc. (VPI) sites, the VPI Connector (VPC), and the Boston Living Center. VPI are low-barrier locations providing services such as harm reduction, testing for sexually transmitted infections, and support groups. We initially recruited from the VPC, a site focused on individuals who identify as women, to develop the recruitment approach. We later added the Boston Living Center, which offers services to all genders, as an additional site.

We recruited study participants between April and July 2023. Inclusion criteria were as follows: (1) age ≥ 18 years; (2) person who used drugs within 6 months by self-report (excluding cannabis); (3) persons without HIV by self-report; and (4) English speaking. Individuals on PrEP were excluded.

Participants completed an assessment that included demographics, substance use, sexual and mental health history, and satisfaction with the approach. Participants also completed the acceptability of intervention measure, the feasibility of intervention measure [7], and received a $25 gift card for compensation.

The research assistant (RA) offered participants rapid saliva-based HIVST kits, OraQuick. During the 20-minute processing time, the RA administered study-related assessments. The RA also provided HIV- and PrEP-related resources. Individuals who tested positive were offered confirmatory testing and linkage to HIV care.

We used descriptive statistics to characterize participants. We present information on participants who completed HIVST, test results, as well as knowledge and interest in PrEP. We also present results from a 5-point Likert scale capturing the extent to which participants agreed with feasibility and acceptability statements. Higher scores indicated greater feasibility and acceptability. All analyses were performed using Excel (Microsoft, Redmond, WA).

## RESULTS

**Figure 1:**
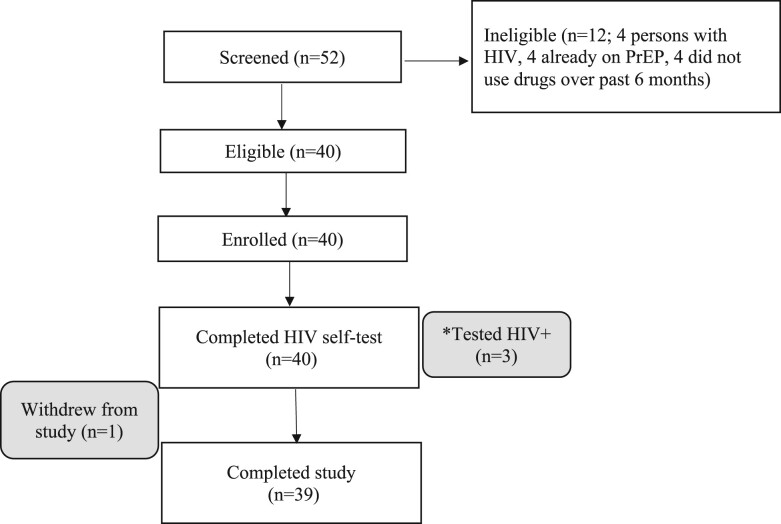
Study flow. *Two individuals with positive tests accepted confirmatory testing and were linked to HIV care; 1 individual was not ready to engage in HIV care.

Among 52 individuals screened, 40 (77%) individuals were enrolled and 39 completed the study (Figure 1). Twelve (23%) individuals were excluded (4 with HIV, 4 already on PrEP, and 4 who had not used drugs in the past 6 months). Thirty-seven (93%) were recruited at VPC, whereas 3 (8%) participants enrolled at Boston Living Center. Of those enrolled, the mean age (standard deviation [SD]) was 37 [8] years; 68% White, 18% Black, 18% Latino, 5% Indigenous, 5% other; 85% female, 5% male, 5% nonbinary; 5% other; 43% (17/40) shared injection equipment in their lifetime, and 68% (27/40) injected substances in the past 6 months (Table 1). Thirty-seven (93%) participants had unstable housing, including 22 (55%) living on the street and 7 (18%) in an overnight shelter. Thirty-two (80%) participants reported being unemployed and 8 (20%) were on disability. Thirty-eight (95%) participants were on MassHealth, Massachusetts Medicaid.

**Table 1.** Participant Demographics (n = 40)

Characteristics	Overall
Race/ethnicity* (n)	
White	27
Black or African American	7
American Indian or Alaska Native	2
Latino/Hispanic	7
Other	2
Gender (n, %)	
Male	2 (5)
Female	34 (85)
Nonbinary	2 (5)
Other	2 (5)
Transgender (n, %)	
Yes	5 (13)
No	35 (87)
Sexual orientation (n, %)	
Heterosexual	23 (58)
Gay	2 (5)
Bisexual	12 (30)
Pansexual	2 (5)
Other	1 (2)
Educational attainment (n, %)	
Some high school	11 (27)
Vocational/trade school	1 (2)
Completed high school or GED	23 (58)
Some college	5 (13)
Housing status, past 6 mo (n, %)	
House or apartment	3 (8)
Shelter or street	29 (73)
Residential treatment facility	2 (5)
Group housing	3 (8)
Jail or prison	2 (5)
Other	1 (2)
Employment* (n)	
Unemployed	32
Disabled	13

*Participants were able to select all that applied.

Regarding risk behavior in the past 6 months, 26 (65%) participants reported daily heroin use through intravenous injection, 27 (68%) participants used daily fentanyl, and 17 (70%) participants were overdose survivors. Sixty-three percent of individuals reported condomless sex. Twenty-six participants reported testing for sexually transmitted infections in the past year and 13 had a positive result.

Before the study, 4 (10%) participants had previously used an HIVST, 35 (90%) had been tested with laboratory-based testing, and 16 (41%) had undergone point-of-care rapid testing other than HIVST. We identified a high-test positivity rate with 3 (8%) participants recording newly positive HIVST. The study team offered confirmatory testing and linkage to care to all individuals with positive testing. Two of 3 participants agreed to have confirmatory testing and linkage to care and were initiated on antiretrovirals by their new HIV care clinicians. One of the participants was not yet ready for these steps, but the VPI has continued supporting the individual and will provide assistance when they are ready. Of all participants enrolled, 31 (80%) were aware of PrEP and 32 (82%) knew that it is recommended by the Centers for Disease Control and Prevention for individuals who inject drugs and do not have HIV. Thirty-two (82%) participants were interested in receiving PrEP. Of those interested, 56% would prefer using an injectable form of PrEP.

Overall, 37 (95%) participants reported being satisfied or very satisfied with using HIVST. Thirty-eight (97%) participants reported being satisfied or very satisfied in their ability to perform and receive an accurate result. Of the participants who completed the exit interview, 39 (100%) reported they would recommend HIVST to others, and 36 (92%) participants agreed that HIVST was more convenient than other HIV tests. Thirty-nine (100%) agreed that they liked HIVST and 39 (100%) also agreed that the test was easy to use.

Participants used a 5-point Likert scale to rate the extent to which they agreed with each feasibility and acceptability statement. We assessed the intervention as feasible with 4.4/5.0 rating (SD = 0.5) on feasibility of intervention measure and acceptable with 4.4/5.0 rating (SD = 0.5) on acceptability of intervention measure.

## DISCUSSION

We identified a test positivity rate of 8% with 3 new HIV cases in a study providing access to rapid saliva HIVST and educational resources on HIV PrEP at community-based harm reduction centers. We also recorded high acceptability and feasibility for HIVST as all participants indicated the test was easy-to-use and they would recommend it to others. Although HIVST was described as a potential “game-changer” when first approved by the Food and Drug Administration, this outlook has yet to be achieved. Nevertheless, increased awareness and comfort with self-testing during the COVID-19 pandemic could potentially lead to HIVST contributing to 2 EHE pillars: rapid diagnosis and HIV cluster identification. We provided rapid diagnosis to individuals with HIV whose infection would have remained unidentified and show the value of collaborating with community-based centers. This approach could provide better access to individuals who would have remained undiagnosed if not for our program. Notably, the majority of participants (82%) were interested in PrEP. Access to HIVST could also facilitate the initiation of PrEP-related care when paired with appropriate resources. Our findings also underscore important factors to consider with future iterations of the program. One of 3 individuals who were newly positive by HIVST was not yet ready to consider confirmatory testing and linkage to care. It is notable that they will continue to be supported by the community-based organization until they are ready to access HIV-related care.

Although the majority of prior studies on HIVST were performed outside of the United States or focused on men who have sex with men, a recent publication on persons who use drugs in the United States showed that HIVST was acceptable when provided at a syringe services program [8, 9]. In the current study, we expand on prior studies by using validated measures to assess acceptability and feasibility. We also show a higher test positivity rate and more enthusiasm for PrEP among participants, including injectable forms, which could inform efforts to address barriers to PrEP uptake among persons who use drugs. We also demonstrate how HIVST could contribute to reaching EHE goals. Within this framework, HIVST could assist with diagnosing people with HIV so that they could be linked to care while also identifying individuals who might be eligible for PrEP.

There are limitations to our study, including the sample size and the inclusion of mainly cisgender women, which might limit generalizability, but we report information on acceptability and feasibility that could inform future studies. Our results also add to the literature by focusing on the potential pairing of HIVST with PrEP-related resources to increase the uptake of effective HIV prevention methods within the community. Furthermore, although we asked whether individuals had previously been tested for HIV, we did not collect information on when they were last tested. Additionally, assessments were administered while tests were running instead of after results were available.

## CONCLUSION

HIVST identified new HIV cases in the community, demonstrating its role as a potential “game-changer” addressing 2 pivotal EHE pillars: rapid diagnosis and identification of HIV clusters. HIVST also offers privacy, enhances autonomy, and meets individuals where they are. Future studies should focus on using HIVST to improve initiation and retention on HIV PrEP as well as same-day HIV treatment.

## Notes

***Financial support***: The authors thank study participants and our research team for their contributions to this work. We also thank Benjamin Linas, MD, MPH, for editorial input and Sarah Miller for copyediting support. This work was supported by the National Institutes of Health (NIDA K23DA044085, NIDA K23 DA044085-03S1, NIDA R01 DA058367, NIAID 5P30AI042853 to S.A.A) and a Boston University School of Medicine Department of Medicine Evans Career Investment and Evans Junior Faculty Merit Awards to S.A.A. The content is solely the responsibility of the authors and does not necessarily represent the official views of National Institutes of Health.

***Patient consent statement***. The patient's written consent was obtained. The study was approved by the Boston University Medical Campus institutional review board and a Certificate of Confidentiality was obtained from the NIH.
